# Common Hepatic Duct Mixed Adenoneuroendocrine Carcinoma Masquerading as Cholangiocarcinoma

**DOI:** 10.1155/2016/4827050

**Published:** 2016-06-07

**Authors:** Sali Priyanka Akhilesh, Yadav Kamal Sunder, Tampi Chandralekha, Parikh Samir, Wagle Prasad Kashinath

**Affiliations:** ^1^Department of Surgical Gastroenterology, Lilavati Hospital and Research Centre, A-791, Bandra Reclamation, Bandra West, Mumbai 400 050, India; ^2^Department of Pathology, Lilavati Hospital and Research Centre, A-791, Bandra Reclamation, Bandra West, Mumbai 400 050, India; ^3^Department of Medical Gastroenterology, Lilavati Hospital and Research Centre, A-791, Bandra Reclamation, Bandra West, Mumbai 400 050, India

## Abstract

Bile duct mixed adenoneuroendocrine carcinoma (MANEC) is a rare entity. It is defined as having mixed elements of both neuroendocrine tumors (NET) and an adenocarcinoma element, the lesser component forming at least 30% of the tumor. It is a subtype of neuroendocrine carcinoma (NEC) showing both gland-forming epithelial tumor cells and neuroendocrine cells. It is generally misdiagnosed as cholangiocarcinoma on imaging studies. The preoperative pathological workup from the endoscopic retrograde cholangiography brush cytology usually misses the NET/NEC component since it often lies deeper in the tumor. However, it is reported that it is the NEC component that defines the prognosis of the tumor; hence, it is vital to identify the NEC component. We present a rare case of common hepatic duct (CHD) MANEC that was preoperatively misdiagnosed as cholangiocarcinoma.

## 1. Introduction

The commonest malignant cause of a bile duct stricture is cholangiocarcinoma, with mixed adenoneuroendocrine carcinoma (MANEC) being a rare cause. MANEC is defined as having mixed neuroendocrine tumor/carcinoma (NET/NEC) and an adenocarcinoma element with each being at least 30% of the specimen. Cholangiocarcinoma is suspected based on imaging studies. MANEC mimics cholangiocarcinoma on imaging. Diagnosis is confirmed only on histopathological examination of surgically resected specimen. As only a few cases have been reported thus far, there is paucity of literature regarding its natural history, prognosis, and treatment. We present a case of common hepatic duct (CHD) MANEC that was initially diagnosed and surgically treated as cholangiocarcinoma.

## 2. Case Report

**Figure 1:**
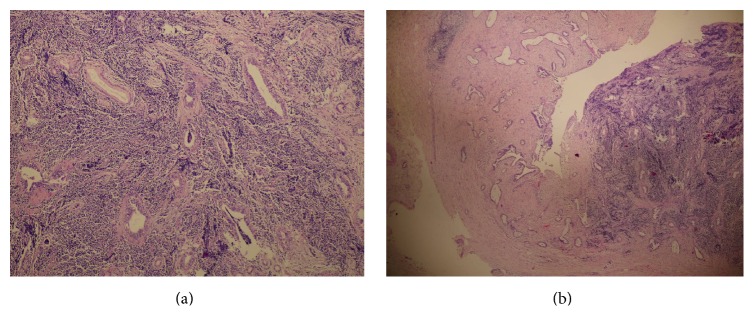
(a) H & E staining showing both NEC and adenocarcinoma component. (b) Scanner view of MANEC in the bile duct (40x).

**Figure 2:**
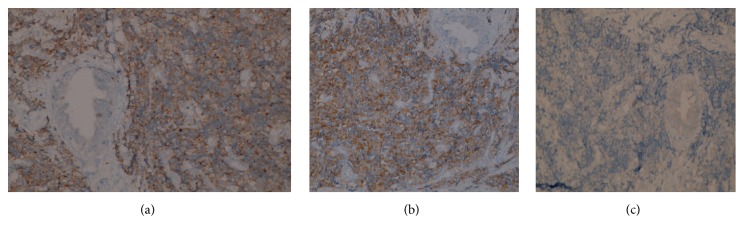
(a) IHC with synaptophysin: strongly positive. (b) IHC with CD 56: strongly positive in the NEC component. (c) IHC with chromogranin: negative.

**Figure 3:**
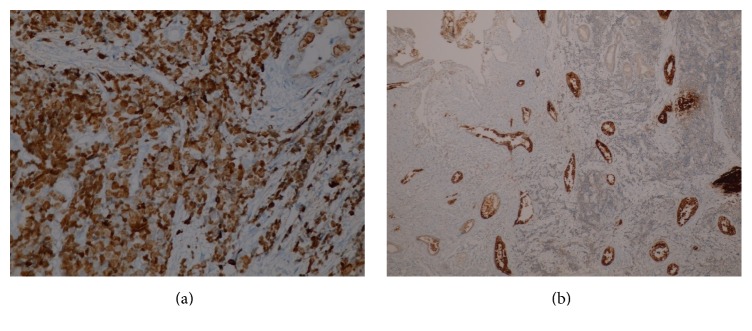
(a) IHC of MANEC with Ki67 400x: 90% in the NEC component and lesser in the adenocarcinoma component. (b) IHC of MANEC with CEA 100x highlights the adenocarcinoma component.

A 76-year-old diabetic and hypertensive gentleman presented with painless progressive jaundice with pruritus 20 days ago. He had lost 11 kilograms in two months. Physical examination was unremarkable except for icterus. On investigations, his total bilirubin was 11.91 mg/dL with the direct component being 8.70, alkaline phosphatase 190 U/L, GGTP 1109 U/L, SGPT 302 IU/L, and SGOT 277 IU/L. Ultrasonography showed dilated intrahepatic biliary radicles (IHBR). Computerized Tomography (CT) revealed dilated CHD with an enhancing 1 cm lesion at the level of the cystic duct, extending to the CHD associated with IHBR, suggestive of cholangiocarcinoma. CA 19-9 and CEA were normal. This was followed by an endoscopic retrograde air cholangiogram that confirmed a CHD stricture of about 1 cm. Brush cytology showed atypical ductal cells that were suspicious but not confirmatory for malignancy. No stent was inserted. Intraoperatively, a lesion was identified in the CHD at the level of the cystic duct extending upwards; the rest of the bile duct felt normal. Radical excision of the involved bile duct from hilum to its intrapancreatic portion was done with Roux-en-Y hepaticojejunostomy and cholecystectomy was added. Frozen section confirmed malignancy and a diagnosis of MANEC was offered. Histopathology showed a MANEC with free margins (Figures 1(a) and 1(b)). The excised duct measured 4 cm in length, with a raised button like lesion measuring 1.4 × 0.8 cm and 0.5 cm thick with narrowing of the duct lumen. The lesion showed predominantly NEC (70%) admixed with invasive ductal adenocarcinoma (30%), thus representing MANEC. Perineural and intraneural invasion were seen. The rest of the bile duct showed mild hyperplasia to low grade dysplasia. The cystic duct only showed hyperplasia with no malignancy. The NEC component (70%) was small cell, grade III, and positive for synaptophysin (Figure 2(a)) and CD56 (Figure 2(b)). It was negative for chromogranin (Figure 2(c)). Cytokeratin cocktail (CK AE1 and CK AE3) was strongly positive in adenocarcinoma (moderately differentiated) component and weakly positive in NEC component (Figure 3(a)). CEA was positive only in the adenocarcinoma component (Figure 3(b)). The Ki 67 was 90% in NEC component (Figure 3(b)). Perineural invasion was positive. No metastasis was seen in the periportal and periductal nodes. The TNM stage was pT2pN0 (IB). Postoperatively, patient recuperated well. No adjuvant therapy was given.

## 3. Discussion and Review of Literature

MANEC usually comprises two components: a variable grade of differentiated adenocarcinoma and a neuroendocrine component with each component being at least 30% of the tumor [1]. In the bile duct, the adenocarcinoma component usually contains gland-forming or signet ring carcinoma. The neuroendocrine components show small or large neuroendocrine cells. According to the WHO classification (2010), neuroendocrine neoplasms in the digestive system were categorized into NET G1 (carcinoid, mitotic count of <2 per 10 HPF, and/or ≤2% Ki67 index); NET G2 (mitotic count 2–20 per 10 HPF and/or 3–20% Ki67 index); NET G3 (neuroendocrine carcinoma, mitotic count of >20 per 10 HPF, and/or >20% Ki67 index); and MANEC [2].

Huang et al. [1] proposed that since the two components originate from multipotential stem cells, biphenotypic differentiation could occur after carcinogenesis is triggered. Harada et al. [3] concluded that adenocarcinoma element was mostly found on the tumor surface whereas the NEC component is in the deeper tissues infiltrating the stromal and vascular tissues and lymph nodes. These patients generally present with painless progressive jaundice and the imaging studies frequently suggest cholangiocarcinoma. If deemed resectable, surgery is the treatment of choice. However, MANEC is seldom diagnosed preoperatively due to paucity of tissue obtained from the endoscopic retrograde cholangiopancreatography (ERCP) brush cytology. The natural history of these tumors is still under debate with some reporting the NEC element showing more aggressive behavior whereas others concluded that if NEC component is well differentiated, prognosis depended on the adenocarcinoma component [4–6]. However, the NEC component is said to have a greater effect on prognosis. MANEC with NEC component being a large cell type is more aggressive than small cell NEC [7]. Moreover, extrahepatic bile duct MANECs are extremely rare [8] with only few cases reported so far. They are usually diagnosed as only NEC or adenocarcinoma. Diagnosis is often missed on brush cytology on ERCP due to NEC component being either found only in the deeper tissues or embedded in the adenocarcinoma element. Hence, most cases are misdiagnosed as cholangiocarcinoma on ERCP brush cytology [7]. Surgery is the treatment of choice in resectable tumors with chemotherapy, radiotherapy, and somatostatin being used as adjuvant therapies. The more aggressive component of MANEC would define the modality of therapy.

## 4. Conclusion

CHD MANEC is a rare tumor with the diagnosis being obtained only on histopathological diagnosis of the surgically resected specimen. It is necessary to identify the NEC component (large/small cell and grade) through immunological studies to determine the aggressive behavior of tumor and thus predict the best modality of treatment and prognosis.

## References

[1] Huang Z., Xiao W.-D., Li Y., Huang S., Cai J., Ao J. (2015). Mixed adenoneuroendocrine carcinoma of the ampulla: two case reports. *World Journal of Gastroenterology*.

[2] Rindi G., Petrone G., Inzani F. (2014). The 2010 WHO classification of digestive neuroendocrine neoplasms: a critical appraisal four years after its introduction. *Endocrine Pathology*.

[3] Harada K., Sato Y., Ikeda H. (2012). Clinicopathologic study of mixed adenoneuroendocrine carcinomas of hepatobiliary organs. *Virchows Archiv*.

[4] Makino A., Serra S., Chetty R. (2006). Composite adenocarcinoma and large cell neuroendocrine carcinoma of the rectum. *Virchows Archiv*.

[5] Kim T.-Y., Chae H.-D. (2011). Composite neuroendocrine carcinoma with adenocarcinoma of the stomach misdiagnosed as a giant submucosal tumor. *Journal of Gastric Cancer*.

[6] Volante M., Rindi G., Papotti M. (2006). The grey zone between pure (neuro)endocrine and non-(neuro)endocrine tumours: a comment on concepts and classification of mixed exocrine-endocrine neoplasms. *Virchows Archiv*.

[7] Lee S. W., Lee I. S., Cho Y. K. (2014). A case of mixed adenoneuroendocrine carcinoma of the common bile duct: initially diagnosed as cholangiocarcinoma. *Korean Journal of Pathology*.

[8] Sato K., Waseda R., Tatsuzawa Y. (2006). Composite large cell neuroendocrine carcinoma and adenocarcinoma of the common bile duct. *Journal of Clinical Pathology*.

